# Pregnancy and umbilical cord pathology: structural and functional parameters of the umbilical cord

**DOI:** 10.25122/jml-2023-0025

**Published:** 2023-08

**Authors:** Bohdan Ihorovych Dubetskyi, Oksana Mykhailivna Makarchuk, Oksana Yaroslavivna Zhurakivska, Mariiana Ivanivna Rymarchuk, Oksana Anatoliivna Andriets, Tetiana Liubomyrivna Lenchuk, Kseniia Marianivna Delva, Madalina Piron-Dumitrascu, Oksana Valerianivna Bakun

**Affiliations:** 1 Department of Obstetrics and Gynecology named after I. Lanovyi, Ivano-Frankivsk National Medical University, Ivano-Frankivsk, Ukraine; 2 Department of Human Anatomy, Ivano-Frankivsk National Medical University, Ivano-Frankivsk, Ukraine.; 3 Department of Obstetrics and Gynecology, Bukovinian State Medical University, Chernivtsi, Ukraine.; 4 Department of Radiology and Radiation Medicine, Ivano-Frankivsk National Medical University, Ivano-Frankivsk, Ukraine.; 5 Private Clinic Leleka Medical Practice, Ivano-Frankivsk, Ukraine.; 6 Department of Obstetrics and Gynecology, Carol Davila University of Medicine and Pharmacy, Bucharest, Romania

**Keywords:** pregnancy, umbilical cord pathology, placental dysfunction, single umbilical artery, velamentous cord insertion, umbilical vein and arteries histostructure, perinatal consequences

## Abstract

Scientific research in the field of physiology and pathology of the umbilical cord is quite limited and imperfect. The purpose of the study was to evaluate the histological architecture of the pathological umbilical cord and investigate the relationship between the main parameters and placental postnatal macromorphometric characteristics, which serve as a reflection of placental dysfunction. Four groups of patients were included, each undergoing a postnatal histological and topographic examination of the umbilical cord: Wharton’s jelly edema (10 samples), velamentous cord insertion (10 samples), single umbilical artery (10 samples), and physiological pregnancy (10 samples). Compared to the control group, all newborn groups exhibited changes in umbilical vessel morphology, characterized by an increased Wagenworth index and a decreased Kernohan index. The functional indices of the umbilical vessels were found to be most severely affected in cases of Wharton's jelly edema. In cases of single umbilical artery, the changes in vascular functional parameters indicated their compensatory remodeling with the highest Wagenworth and Kernohan indices of the umbilical vein. Deviation from the normal average placental weight was observed in cases of Wharton's jelly volume pathology or velamentous cord insertion. However, in the case of a single umbilical artery, there were no significant deviations in the macromorphometry of the placenta.

## INTRODUCTION

The extent and specifics of how pathological factors influence the progression of pregnancy and its outcomes largely rely on the preservation of compensatory and adaptive mechanisms within the utero-fetoplacental system. The functional parameters of this system determine the level of disruption in fetal condition [1-5]. Abnormalities in the structure and location of the umbilical cord are associated with intrauterine distress, asphyxia, stillbirth, increased risk of congenital malformations, fetal aneuploidy, intrauterine growth restriction, and complications such as gestational diabetes and preeclampsia [1, 3-5]. Numerous studies have suggested an indirect association between placental dysfunction and hypoxic conditions, leading to morphometric changes in extraembryonic structures [6-8]. For example, fetal growth retardation syndrome is associated with a deficiency in the development of terminal plates, leading to tissue oxygenation disturbances. Decreased vascularization intensity of the villi and dystrophic changes in the stroma can disrupt the interaction between arterial and venous channels in the placental tissue, which may prompt the use of antenatal ultrasound of placental parameters to predict pregnancy outcomes [9-11]. However, the causes of umbilical cord pathology and its influence on perinatal consequences are not well understood despite their association with congenital malformations, chromosomal abnormalities, perinatal complications, and reproductive losses [4, 5, 12-15]. According to literature reports, umbilical cord pathology is a direct cause of stillbirth, early infant mortality, and morbidity of newborns in 21-65% of cases, and its frequency in the population ranges from 4.8 to 38.4% [1, 4, 14-17]. However, despite the critical importance of the umbilical cord in fetal development, scientific research in its physiology and pathology remains limited and imperfect. This knowledge gap is particularly concerning given the significant prevalence of hypoxic-ischemic damage to the central nervous system of newborns resulting from umbilical cord pathology. This highlights the urgent need to develop basic monitoring principles and transparent criteria for making informed decisions regarding obstetric management in pregnancies affected by umbilical cord abnormalities.

Studies conducted by Kadivar M. and other researchers have demonstrated that gross morphological characteristics, such as an increase in the diameter of the umbilical cord and edema, are more frequently observed among parturients with pregnancy complications, particularly gestational diabetes. However, early diagnosis, implementation of an effective diagnostic strategy, and optimal glycemic control have shown promising results in mitigating the severity of histomorphological changes in the umbilical cord among these women [1, 13].

The umbilical cord plays a crucial role as a structural unit within the fetoplacental complex, functioning autonomously with unique structural and functional characteristics [1, 17-19]. In many prenatal ultrasound departments, important sonographic findings such as true knots in the umbilical cord are often overlooked and not documented, and patients are not informed about this potentially life-threatening condition, which carries a significant risk of stillbirth [18]. To obtain an accurate assessment of umbilical cord blood flow functionality, it is essential to evaluate a comprehensive set of diverse organometric and histometric parameters that have not received sufficient attention from the scientific community. This assessment should also consider the interdependence between the vascular and mesenchymal components of the umbilical cord, considering specific developmental anomalies and the severity of placental dysfunction. In addition, it is essential to investigate the mechanisms underlying changes in the volume and velocity of umbilical cord blood flow, which hold significant scientific and practical value [9-11, 20].

The umbilical cord is the only connection between the mother and the fetus, facilitating the transport of respiratory gases, nutrients, and metabolites. It is through this lifeline that proper fetal psychomotor development occurs [20, 21]. Therefore, it is necessary to assess the anatomy of the umbilical cord during ultrasound examination in the first trimester. This includes sonographically confirming the correct number of umbilical cord vessels and carefully evaluating the insertion site. Diagnostic technologies such as sonography (including color Doppler mapping), Doppler ultrasound, and cardiotocography have proven valuable in diagnosing umbilical cord pathology. However, to achieve a more comprehensive and informed approach to diagnosis, decision-making, and selection of obstetric strategies, it is crucial to have a thorough understanding of the underlying pathological processes occurring in the placental and umbilical cord tissue in these conditions. This understanding serves as the basis for the relevance and importance of conducting this scientific research.

This study aimed to evaluate the structural and stromal architectonics of the umbilical cord in cases of pathological conditions and investigate the relationship between the main organometric parameters and the postnatal macromorphometric characteristics of the placenta, which could serve as indicators of placental dysfunction.

## MATERIALS AND METHODS

### Study design and participants

This study was part of a larger research project titled "Development of diagnostic tactics and pathogenetic substantiation of effective methods of preservation and restoration of reproductive potential and improvement of parameters of women's life quality in obstetric and gynecologic pathology" (state registration number 0121U109269, implementation dates 2021-2026).

The study followed a randomized, controlled, and prospective design. The main group consisted of 90 pregnant women diagnosed with umbilical cord pathology, including 34 with Wharton's jelly edema, 29 with velamentous cord insertion, and 27 with a single umbilical artery. Exclusion criteria included acute infectious diseases during pregnancy, fetal malformations, fetopathy associated with dysmetabolic or immune disorders, uterine malformations, multiple pregnancies, severe somatic diseases, and patient refusal to participate. The control group included 30 healthy women with a physiological course of pregnancy and without umbilical cord pathologies who gave birth to healthy full-term infants. The age of the patients ranged from 18 to 44 years, with an average of 37.8±2.5 years.

### Data collection and measurements

All pregnant women in the main group underwent comprehensive ultrasound examinations as part of the antenatal pregnancy monitoring program. These examinations were conducted between 11 to 14 weeks of gestation at expert-level facilities, including the family planning offices of the Ivano-Frankivsk Regional Perinatal Center of the Ivano-Frankivsk Regional Council and the clinic Leleka Medical Practice in Ivano-Frankivsk.

The ultrasound examinations were performed in a dedicated prenatal diagnostics room using the Voluson E8 device with a transducer operating at frequencies of 3.5 and 5.0 MHz according to the generally accepted transabdominal and transvaginal technique. The ultrasound assessment of the umbilical cord focused on several key parameters, including the site of insertion of the umbilical cord to the placenta, the place of attachment of the umbilical cord to the anterior abdominal wall of the fetus, the number of arteries in the umbilical cord, and any pathological changes observed, such as alteration in the volume of Wharton’s jelly. To assess the fetal state in the presence of umbilical cord pathology and diagnose placental dysfunction, objective methods were employed, including cardiotocography, ultrasound, and dopplerometry. Patients were included in the study after diagnosing umbilical cord pathology and signing the informed consent.

In the second stage of the study, a postnatal organometric and histo-topographic examination of the umbilical cord and postnatal macromorphometry of the placenta were conducted for women in the three main groups. Group 1 consisted of 10 parturients with verified pathology of the Wharton's jelly volume, Group 2 included 10 parturients with pathology of umbilical cord insertion (velamentous cord insertion), and Group 3 comprised 10 parturients with a vascular anomaly of the umbilical cord (a single umbilical artery). In addition, for the purpose of comparison, the umbilical cords and placentas of 18 patients in the control group (K) were studied.

The organometric method involved measuring the weight of the placenta, its dimensions, the average arithmetic thickness, the volume of the organ and the area of the maternal surface of the placenta, as well as the diameter and thickness of the umbilical cord, its characteristics (length, tortuosity, “thinness” index, insertion features) and human fetal-placental weight ratio, which is calculated by dividing the weight of the placenta by the weight of the newborn [22]. The following parameters were chosen as the most informative organometric parameters of the umbilical cord characteristics: linear mass unit (LMU g/cm), volumetric mass unit (VMU g/cm^3^), and umbilical cord diameter (UCD cm).

The external examination of the placenta and umbilical cord was performed systematically. The fetal surface, membranes, umbilical cord, fragments, and maternal surface were sequentially evaluated. Cross-sections of different parts of the placental disc were examined to assess tissue condition, shape, color, characteristics of pathological foci, branching nature, and degree of vascular tortuosity. The mass of the placenta was determined after separating the umbilical cord and fetal membranes, the diameters of the placenta were measured from the side of the maternal surface, and the thickness was estimated by piercing through with a needle with marks in the areas of insertion of the umbilical cord and the marginal zone. The weight of the umbilical cord and the umbilical coiling index (UCI – the ratio between the number of turns and its length, where the average value (median) is 1 turn per 5 cm or 0.2±0.1 turns per 1 cm) were determined. According to the literature, the normal distribution of the UCI was determined: 5 percentiles – 0.06; 50 percentiles – 0.18; 90 percentiles was 0.37. UCI was considered the most significant parameter of the umbilical cord, associated with high perinatal risk [1].

The morphological study was conducted at the Educational and Scientific Laboratory of Morphological Analysis at the Ivano-Frankivsk National Medical University of the Ministry of Health of Ukraine. Pieces of the umbilical cord were fixed for 24 hours in a 10% neutral buffered formaldehyde solution. The material was then prepared as blocks and stained with hematoxylin and eosin (H&E) following the standard procedure [22]. Light microscopy was performed using a Leica DM 750 microscope with ×10, ×20, ×40, and x100 lenses. Photo documentation was done using a digital CCD camera with a resolution of 1200x1600 saved in JPG format. Photographs of histological sections were used for morphometric studies of the umbilical cord. To measure the metric characteristics of the umbilical cord vessels, NIH USA Image Jsoftware (USA) was used in manual mode, considering magnifications. The indicators of the area of the profile field of the umbilical artery and vein, their wall, and lumen were determined. To assess the functional state of vessels, the Wagenworth index was determined – the ratio of the area of the vessel to the area of its lumen, and the Kernoghan index (KI) - the ratio of the area of the lumen of the vessel to its total area [22, 23].

### Statistical analysis

Statistical analysis was performed using the Statistica 7.0 software package. Descriptive statistics, including mean values and standard deviations, were calculated for quantitative variables. Student's t-test was used to determine differences in quantitative indicators between groups, while the χ^2^ test was used for qualitative indicators. Statistical significance was set at p<0.05. The study employed a random sampling method.

## RESULTS

### Prevalence of umbilical cord pathologies

Umbilical cord pathology or abnormality of its placental insertion was diagnosed at different stages of pregnancy and postpartum. Among the cases studied, 19 (21.1%) were diagnosed between 12-22 weeks of gestation, 42 (46.7%) were diagnosed before full-term pregnancy, and 29 observations (32.2%) were made after childbirth. In approximately half of the observations (51.1%), a combination of umbilical cord characteristics was identified. This combination included Wharton's jelly absence along with either velamentous cord insertion or a combination of a "thin" umbilical cord with a single umbilical artery (Table 1). Furthermore, the assessment of various factors such as age, gynecological and somatic morbidity, and reproductive potential made it possible to establish reliable deviations in parameters such as nicotine addiction, older age of first-time mothers, overweight and obesity in case of umbilical cord pathology, as well as an increase in the proportion of such complications as prematurity, preeclampsia, and placental dysfunction. In nearly one-fifth of the cases, pregnancy occurred after using the in vitro fertilization (IVF) program.

**Table 1:** Characteristics of the studied groups, n=120, absolute number, %

Indicator	Main group, n=90	Control group, n=30
Combination of various types of umbilical cord pathology	46 – 51.1 *	-
Harmful habits (smoking)	39 – 43.3*	7 – 23.3
Mother’s age >35 years	53 – 58.9*	10 – 33.3
Birth parity (first birth)	31 – 34.4	6 – 20.0
Using IVF programs	22 – 24.4*	2 – 6.67
Overweight and obesity	31 – 34.4*	4 – 13.3
Gestational diabetes	13 – 13.4*	1 – 3.3
Change in amniotic fluid index (oligohydramnios)	41 – 45.6	6 – 20.0
Premature pregnancy	22 – 24.4*	1 – 3.3
Preeclampsia	34 – 37.8*	3 – 10.0
Placental dysfunction	40 – 44.4*	5 – 16.7
Birth of a low-weight fetus	17 – 18.9*	1 – 3.3
Pathology of the early neonatal period	20 – 22.2*	-
Abdominal delivery	29 – 32.2*	4 – 13.3

* – the difference is significant against the data of the control group, p<0.05

### Clinical course of pregnancy and childbirth

The analytical assessment of the clinical course of pregnancy and childbirth in patients with umbilical cord pathology revealed several significant findings. There was a significant rate of prematurity (24.4%), change in amniotic fluid index (a decrease in the amniotic index – oligohydramnios) (45.6%), preeclampsia (37.8%), placental dysfunction (44.4 %), birth of low-weight fetus (18.9%), pathologies of the early neonatal period (22.2%), and an increase in the proportion of abdominal deliveries (32.2%). During the antenatal observation stage, the following main pathological cardiotocographic signs were also identified in the main groups. Basal tachycardia was observed in 39 cases (43.3%), a decrease in the number of accelerations in 40 cases (44.4%), a decrease in the variability of the basal rhythm in 40 cases (44.4%) ), an undulating type of basal rhythm variability in 51 cases (56.8%), the appearance of acceleration-deceleration-acceleration complexes in 37 cases (41.1%), variable decelerations in 32 cases (35.6%), and wave-like sinusoidal rhythm in 59 cases (65.6 %).

The results of the Doppler ultrasound evaluation showed a violation of uterine-placental blood flow of 1^st^A degree in 46 cases (51.1%), 1^st^B degree – in 9.5% of patients in Group 1, and 25% of patients in Group 2. Violation of placental-fetal blood circulation (2^nd^ degree) was revealed in 30 (33.3%) pregnant women, and violation of fetal blood circulation – in 14 (15.6%) women, which created prerequisites for urgent delivery. Furthermore, fetal distress syndrome, as indicated by cardiotocography, was observed in 52 instances (57.8%), a significantly higher frequency than among the control group.

### Organometric measurements of the placenta and umbilical cord

At the second stage of the study, organometric measurements were conducted to assess the average weight of placentas. The results showed variations in the average placental weight across different groups: 516.62±4.56 g in Group 1, 382.48±6.42 g in Group 2, 484.82±4.53 g in Group 3, and 449.68±3.48 g in the control group. These findings indicate deviations from the reference norms, particularly in umbilical cord pathology involving Wharton’s jelly volume or pathological cord insertion. Such deviations make the implementation of compensatory and adaptive reactions of the placenta ineffective. However, in the case of a single umbilical artery, reliable deviations in placental weight were not observed.

External examination of such placentas revealed isolated areas of hemorrhages on the maternal surface small, diffusely scattered areas of necrosis and petrification. A certain indirect reflection of the conditions of placentation and the probability of the development of placental dysfunction was the shape of the placenta. Abnormalities in placental shape were more prevalent in the main group, with an oval shape observed in 33 observations (36.7%) compared to only 3 observations (10.0%) in the control group. An abnormal shape was found in 21 patients (23.3%) in the main group, while only 4 cases (13.3%) showed such abnormalities in the control group. The main and intermediate types of vascular branching were also more pronounced in the main group, with 61 cases (67.8%) exhibiting these abnormalities compared to only 4 cases (13.3%) in the control group.

### Postnatal observations of the umbilical cord

During the postnatal stage of the study, several observations were made regarding the umbilical cord. A significant proportion of cases (36.7% and 34.4%, respectively) involved long umbilical cords measuring 70 cm or more, and in one-third of cases, the umbilical cord was found to be wrapped around the neck or other parts of the fetus. Additionally, 13 cases (14.4%) had an extremely short umbilical cord. The specific gravity of the fetal fragment of the umbilical cord was significantly higher compared to the data of the placental fragment (0.96±0.03 g/cm *vs*. 0.62±0.05 g/cm). Furthermore, the average umbilical cord weight ranged from 22.6±0.01 g (primarily in the case of velamentous cord insertion) to 143.4±3.24 g – in the case of an excess of Wharton’s jelly. In more than one-third of the samples, the weight of the umbilical cord was in the range of 32.8±1.12 g to 69.6±3.42 g, which corresponded to the weight parameters of umbilical cords in full-term uncomplicated pregnancies. According to the results of the postnatal measurement of the umbilical coiling index, the parameters corresponding to the reference limits of generally accepted criteria (0.2/cm) were established only in 21 cases (23.33%). During the morphometric analysis of the umbilical cords in Group 1, a probable decrease in the area of their arteries was revealed due to a pronounced decrease in both the area of the artery wall and the area of the artery lumen (Table 2).

**Table 2:** Morphometric characteristics of umbilical arteries (X±SD, mm^2^)

Studied groups	Vessel area	Lumen area	Wall area
**Control group, n=10**	1635.89±110.60	89.90±5.96	1545.99±104.97
**Group 1, n=10**	1410.47±169.04*	14.84±2.90*	1395.64±166.77*
**Group 2, n=10**	2389.49±283.65*^#^	26.73±4.15*^#^	2362.76±280.81*^#^
**Group 3, n=10**	5517.29±618.61*^#β^	21.27±11.91^#^	5496.02±606.75*^#β^

* - the difference is probable against control data, p<0.05

# -the difference is probable against the data of Group 1, p<0.05

β - the difference is probable against the data of Group 2, p<0.05

**Figure 1:**
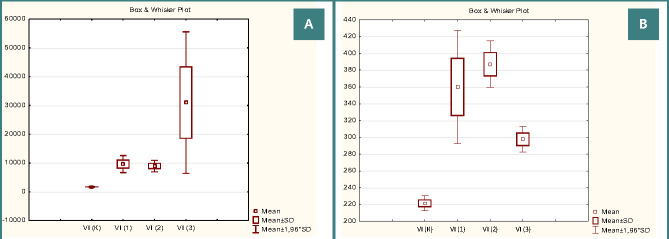
Quantitative changes in the Wagenworth index of the umbilical artery (A) and vein (B)

Compared to the other studied groups, the area of the umbilical arteries and their wall in Group 1 was closest to the control indicators, while the area of the lumen was the smallest. The Wagenworth index, which reflects the permeability of the vessels, was found to be significantly higher in Group 1 (9617.27±1507.77%) compared to the control group (1720.11±40.33%) (p<0.05). This indicates a substantial decrease in the permeability of the umbilical arteries in Group 1 (Fig. 1 a-b).

**Figure 2:**
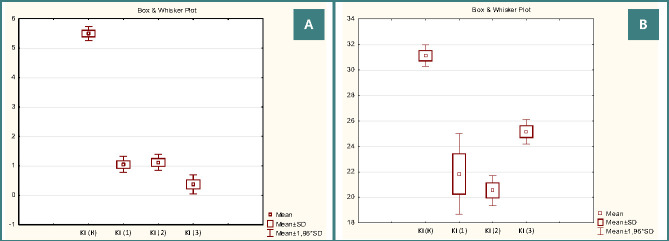
Quantitative changes in the Kernohan index of the umbilical artery (A) and vein (B)

According to morphometric data, the Kernohan index decreased to 1.05±0.14% (in the control group – 5.5±0.12%) (Fig. 2 a-b), which may be an indirect indication of blood flow disturbance in these vessels.

Examining the umbilical vein, a probable thickening of its wall due to decreased lumen with an unchanged vessel area was found (Table 3). Such quantitative reconstruction of the vein led to an increase in the Wagenworth index to 360.01±34.5% (in the control – 221.47±4.47%, p<0.05), while the Kernohan index decreased to 21.83±1, 61% (in the control – 31.11±0.44%, p<0.05) (Fig. 1-2).

**Table 3:** Morphometric characteristics of umbilical veins (X±SD, mm^2^)

Studied groups	Vessel area	Lumen area	Wall area
**Control group, n=10**	3536.41±93.26	1100.50±42.97	2435.91±52.04
**Group 1, n=10**	3739.01±152.62	815.85±60.41*	2923.16±148.15*
**Group 2, n=10**	3931.98±145.52*	808.58±52.77*	3123.39±94.07*^#^
**Group 3, n=10**	4627.37±164.35*^#β^	1163.99±44.51*^#β^	3463.37±127.83*^#β^

* - the difference is probable against control data, p<0.05

# -the difference is probable against the data of Group 1, p<0.05

β - the difference is probable against the data of Group 2, p<0.05

**Figure 3:**
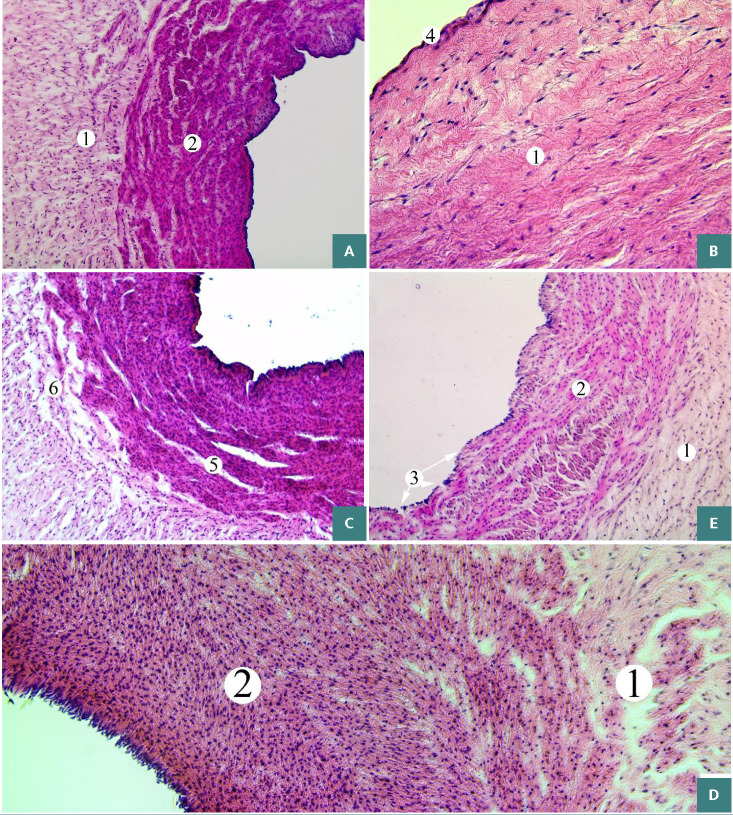
Histological features of the umbilical vein in the control group (A, B), Group 1 (C), Group 2 (D), and Group 3 (E). Hematoxylin-eosin staining. Photomicrographs (a-d), panoramic photomicrograph (e). Magnification: a, c, e)x100; b, d)x200. Designation: 1 – Wharton’s jelly, 2 – tunica media of a vein, 3 – partial desquamation of the endothelium, 4 – amniotic epithelium, 5 – edema and stratification of myocytes in the tunica media of a vein, 6 – edema and cavity formation in Wharton’s jelly.

The histological features of the umbilical vein across groups can be seen in Figure 3 (a-e). The umbilical cord in the control group had a classic structure. At the microscopic level, three vessels (two arteries and one vein) surrounded by Wharton’s jelly were visualized. Two zones could be distinguished in the structure of the latter: the central (more compact one, which directly borders with the outer layer of the vessels) and the peripheral one (characterized by a spongy texture and covered by amniotic epithelium) (Fig. 3 a-b).

The structural parameters of the venous wall in Group 1 were characterized by focal stratification and myolysis of myocytes of the middle membrane (Fig. 3c). At the same time, the histostructure of Wharton’s jelly was less dense due to the swelling of connective tissue fibers, compared to the control group.

**Figure 4:**
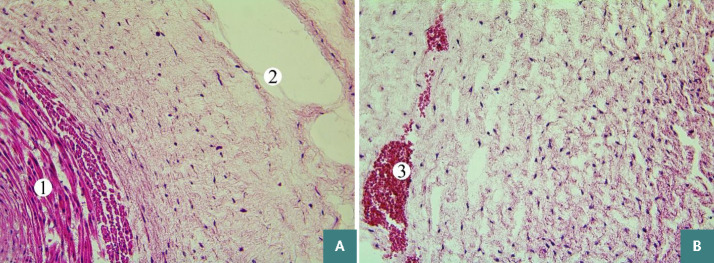
Oedema and stratification of connective tissue fibers of Wharton's jelly in the umbilical cord of newborns in Group 1. Hematoxylin-eosin staining. Photomicrographs. Magnification: A, B) x200. Designation: 1 – myocytes of the umbilical artery, 2 – cavities in Wharton’s jelly, 3 – hemorrhages in the peripheral zone of Wharton’s jelly.

Furthermore, the small and large cavities in Wharton’s jelly over the entire umbilical section area and focal hemorrhages in the peripheral zone were observed (Fig. 4).

In the case of velamentous cord insertion (Group 2), the same type of quantitative rearrangement of the umbilical arteries and veins was noted. As seen in Tables 2 and 3, compared to the control indicators, the area of the umbilical vein and arteries increased due to the increase in the area of their wall, while the area of their lumen probably decreased. This led to an increase in the Wagenworth index in the umbilical vein and arteries by 386.0±14.18% and 8944.12±1024.15%, respectively, and a decrease in the Kernohan index by 20.55±0.60% and 1.12±0.14%, respectively (in all cases p<0.05).

Histological analysis revealed the hypertrophy and proliferation of myocytes leading to the thickening of the muscular layer within arteries and veins, while the number of connective tissue fibers did not differ from the control group (Fig. 3 d). The luminal structure of all umbilical arteries in Groups 1 and 3 was slit-like, differing from the control group, where an oval shape predominated, often filled with formed blood elements.

In newborns from Group 3 with a single umbilical artery, the umbilical cord contained only two vessels, an artery, and a vein. The morphometric analysis indicated that these vessels exhibited the highest thickness across all investigated groups, including the control group. This heightened thickness could be attributed to a substantial increase in the muscular layer of their walls (Tables 2-3). A considerable thickness of the media of umbilical vessels led to an increase in the Wagenworth index and a decrease in the Kernohan index, compared to the control indicators in the umbilical vein – up to 297.64±7.87% and 25.16±0.49%, and in the umbilical artery – up to 31004.52±12533.26% and 0.37±0.16% (in all cases p<0.05).

In the lumen of the umbilical vein, the desquamation of the endothelium was quite frequent (Fig. 3 e). In the umbilical artery, the thickening of the tunica media occurred due to the hypertrophy of myocytes and the growth of collagen fibers, while in the wall of the umbilical vein, a thinned subendothelial elastic layer was clearly visualized.

In all the studied groups of newborns, except for the control group, we observed changes in the umbilical vessels characterized by specific quantitative modifications. However, a common feature among Groups 1, 2, and 3 was the indication of impaired blood flow in these vessels based on the functional indices. Thus, in the case of an excess of Wharton’s jelly in the umbilical cord, the arteries and veins had the smallest area, including the area of the lumen and the wall area, compared to Groups 2 and 3. This difference was observed only in the umbilical arteries compared to the control group. Several authors indicated that the pathology (edema) of Wharton’s jelly leads to compression of the umbilical vessels and, as a result, to disruption of blood supply to the fetus and even death [9, 17], confirmed by our data. Quantitative changes in the vessels of the umbilical cord led to an increase in the Wagenworth index and a decrease in the Kernohan index, compared to the control, which indicates a deterioration in the permeability of these vessels and a violation of blood flow. The small and large hemorrhages in the peripheral parts of Wharton’s jelly indicate damage to the umbilical cord during intrauterine development.

In the umbilical cords with velamentous insertion, an increase in the area of arteries and veins was noted due to the thickening of their walls, which led to an increase in the Wagenworth index (which was the largest in the arteries of all groups) and to a decrease in Kernohan index. Deterioration of blood flow in the vessels of the umbilical cord can lead to fetal growth restriction, which is often diagnosed using ultrasound examination [10, 11, 21].

In newborns from Group 3, the umbilical cord contained two vessels. Notably, the umbilical artery and vein showed significant hypertrophy, with their areas being the largest among all the studied groups. Previous literature suggests that maternal diabetes can lead to the atresia of one of the umbilical arteries [13, 24]. Scientific sources indicate that a change in the number of umbilical vessels can be combined with chromosomal abnormalities [19, 25]. In Group 3, the area of the lumen of the umbilical vein probably did not differ from the control indicators, which contributed to the improvement of blood flow in this vessel. Consequently, Wagenworth and Kernohan indices of the umbilical vein in Group 3 were the highest, compared to samples from Groups 1 and 2, but probably lower than in the control group (Figures 2b, 3b). Such quantitative changes in the functional parameters of the umbilical vessels of newborns in Group 3 indicate their compensatory adaptation to ensure adequate fetal nutrition. Some authors indicate that in 60-90% of cases, one umbilical artery does not affect the course of pregnancy and childbirth, as well as the postnatal development of a newborn [19].

## DISCUSSION

Studies focusing on the precise contribution of pathological umbilical conditions to the development of perinatal complications lack comprehensive systematization.

The available data concerning the feasibility of prenatal diagnosis for each condition are inconsistent. There are no clear algorithms for managing pregnancy and childbirth for every umbilical cord anomaly [4, 16, 18, 19, 26]. Pathologies of the umbilical cord in birth histories are registered only in case of intranatal complications, which makes it difficult to evaluate epidemiological data and makes retrospective studies of many umbilical cord anomalies impossible. In the obstetric community, there is no unified point of view on many pathological conditions of the umbilical cord (a single umbilical artery syndrome, umbilical cord coiling, true umbilical cord knot, etc.), criteria for prenatal and postnatal diagnosis of a “thin” umbilical cord [17-19]. Moreover, an in-depth study and systematization of data concerning the morpho-functional parameters of the umbilical cord can play a crucial role in the early identification of perinatal risk groups. This, in turn, can enhance the accuracy of prognostic assessments related to deviations in the fetal and neonatal health status, ultimately leading to more effective and timely interventions for prevention and treatment [26].

The works by L. Nazarenko and other authors demonstrate that today, much less attention is paid to events in the intranatal period, which have a special risk profile and are characterized by such stressors as acute hypoxia and birth trauma. Various factors play a significant role in the occurrence of hypoxic and traumatic injuries during intrauterine development, and among these, disturbances in placental fetal blood circulation, particularly associated with umbilical cord pathology, are of notable importance [5, 22]. Marginal and velamentous cord insertions are of the greatest clinical importance, examples of blastopathies with a delayed effect or the result of implantation disorders. Randomized studies involving singleton pregnancies in the European female population have revealed a correlation between peripheral and velamentous insertions of the umbilical cord and an increased incidence of miscarriages, premature births, fetal malformations, fetal hypoxia, and intrauterine deaths. Umbilical hypocoiling and hypercoiling (under conditions of pre- or postnatal verification) are associated with adverse perinatal outcomes, although the specific frequency remains uncertain. Hypocoiling is associated with trisomy, preterm birth, antenatal fetal death, increased intranatal complications and operative deliveries due to distress, Apgar score below 7 points at 5 min, velamentous cord insertion, and a single umbilical artery. Hypercoiling is also associated with trisomy but more often with fetal growth retardation, asphyxia, a single umbilical artery, and, according to other data, a high frequency of premature birth. Abnormal tortuosity of the umbilical cord is also the cause of thrombosis of vessels of the chorionic plate, thrombosis of the umbilical vein, and stenosis of the umbilical cord. Preeclampsia and diabetes are factors that increase the likelihood of abnormal tortuosity, as well as fetal growth retardation [22].

The Stillbirth Collaborative Research Network conducted an extensive case-control study spanning from 2006 to 2008, as detailed by Hammad IA *et al*. [27]. Out of 496 stillbirth cases analyzed, which were classified based on INCODE criteria, 19% (95% CI 16-23%) were linked to umbilical cord abnormalities. Forty-five of them (48%) had disorders of fetal microcirculation, 27 (29%) had umbilical cord entrapment, 26 (27%) due to knots, twisting, or stricture, and five (5%) due to umbilical cord prolapse. These observations led to the conclusion that umbilical cord abnormalities constitute a significant risk factor for stillbirth [27].

Many scientists have explored the topic of morphological assessment of the umbilical cord [7, 8, 17, 28]. Within the available sources, we found publications focused on sono-histomorphological examinations of the umbilical cord in cases involving gestational complications, such as preeclampsia, fetal growth retardation syndrome, and others [29, 30].

Thomas MR *et al*. demonstrated that the umbilical cord is the connecting link between the mother and fetus, and its morphology is an indicator of fetal well-being. In their investigation aimed at comparing the structural disparities of the umbilical cord between pregnant women exhibiting normal blood pressure and those with preeclampsia, the researchers conducted evaluations on this subset of patients at full term. The parameters included umbilical cord cross-sectional area (CSA), umbilical vein lumen, umbilical vein wall, umbilical artery lumen, umbilical artery wall, and Wharton’s jelly, including edema of vessel walls and Wharton’s jelly and basement membrane thickening. The mean umbilical cord CSA was significantly higher in the preeclampsia group, whereas the mean umbilical artery lumen CSA was lower. The mean Wharton’s jelly CSA in the case of preeclampsia was also significantly higher, while the umbilical vein lumen CSA, umbilical vein wall CSA, and umbilical artery wall CSA parameters did not show a significant difference. Edema and thickening of the basement membranes were considered significant [30].

Lan Y *et al*. demonstrated the negative impact of the development of preeclampsia not only on the mother’s body but also on the fetus, including impaired fetal development due to a decrease in placental perfusion [29]. Since the vessels of the umbilical cord provide blood supply to the fetus, morphological changes in these vessels can critically affect fetal functions. The authors carried out a morphomicroscopic study of 36 umbilical cords, as well as samples of placental, middle, and fetal segments, assessing wall thickness, lumen diameter, wall-lumen ratio and membrane thickness, smooth muscle area, nuclear area, nuclear density, and the density of the nuclear zone of smooth muscles [29]. It was found that the diameter of the lumen of the umbilical veins gradually decreased, while the wall thickness, membrane thickness, and wall-lumen ratio gradually increased from the placental to the fetal segments of the umbilical vein. Smooth muscle area, nuclear area, nuclear density, and nuclear zone density did not differ significantly from the placental to fetal umbilical vein segments. In cases of preeclampsia, the luminal diameter was smaller, and the wall thickness, membrane thickness, wall-to-lumen ratio, nuclear density, and nuclear zone density were greater. Conversely, no significant difference was observed in the smooth muscle area and nuclear area. Moreover, within the fetal segments of umbilical veins in cases of preeclampsia, correlation analysis showed a negative correlation between the wall-lumen ratio and gestational age, birth weight, and Apgar score. These results indicate that the umbilical vein in cases of preeclampsia has a thickened middle layer due to the proliferation of smooth muscles, which may be an adapted response to hypertension. In general, this study demonstrates that morphological changes in the umbilical veins negatively affect the development of the fetus during preeclampsia [29].

Traditionally, prenatal assessment of the umbilical cord is limited to assessing the number of vessels and blood flow parameters in the umbilical artery. Morphological aspects of umbilical cord pathology were usually studied by pathologists and retrospectively correlated with perinatal outcomes [4, 7, 8, 20]. A number of studies have demonstrated that the altered structure of the umbilical cord can be associated with pathological conditions (preeclampsia, fetal growth retardation, diabetes, fetal death) [4, 29, 30]. Normograms of various components of the umbilical cord have been created to identify thin or swollen umbilical cords, a pathology often associated with fetal abnormalities, excess weight, and gestational diabetes. A reduction or insufficiency of Wharton’s jelly has also been considered a possible cause of intrauterine fetal death, especially when combined with a condition such as a single umbilical artery [14, 15, 19, 28].

## CONCLUSION

Umbilical cord abnormalities represent a significant risk factor for perinatal morbidity and mortality, accounting for up to 22.4% of cases, even when using strict monitoring principles and criteria. An indirect reflection of the conditions of placentation in patients with umbilical cord pathology was the shape of the placenta with a predominance of abnormal types (23.3%), as well as the main and intermediate types of vascular branching (67.8%). Evaluation of average placental weight further indicated significant deviations from normative reference values in cases involving pathology of Wharton’s jelly volume or abnormal umbilical cord insertion. In such instances, the compensatory and adaptive responses of the placenta are ineffective. Conversely, in cases featuring a single umbilical artery, macromorphometric assessments of the placenta did not reveal reliable deviations in the selected parameters. The umbilical cord coiling index corresponded to the reference limits of generally accepted criteria (0.2/cm) only in 23.33% of observations. Pathomorphological changes in the umbilical cord were consistently characterized by an increase in the Wagenworth index and a decrease in the Kernohan index across all three main groups, which is a morphological confirmation of the deterioration of the blood flow of the vascular system (a decrease in their permeability and blood flow). In the case of Wharton’s jelly edema, the indicators of functional indices of vessels were the worst. In the case of a vascular anomaly – a single vessel of the umbilical cord – changes in the functional parameters of the umbilical vessels indicated their compensatory restructuring to ensure normal nutrition of the fetus, which was confirmed by the highest Wagenworth and Kernohan indices of the umbilical vein, compared to Groups 1 and 2.

## Data Availability

Further data is available from the corresponding author on reasonable request.
